# Impressic Acid Attenuates the Lipopolysaccharide-Induced Inflammatory Response by Activating the AMPK/GSK3β/Nrf2 Axis in RAW264.7 Macrophages

**DOI:** 10.3390/ijms22020762

**Published:** 2021-01-14

**Authors:** Gi Ho Lee, Ji Yeon Kim, Sun Woo Jin, Thi Hoa Pham, Jin Song Park, Chae Yeon Kim, Jae Ho Choi, Eun Hee Han, Young Ho Kim, Hye Gwang Jeong

**Affiliations:** 1 College of Pharmacy, Chungnam National University, Daejeon 34134, Korea; ghk1900@cnu.ac.kr (G.H.L.); jykim525@o.cnu.ac.kr (J.Y.K.); mpassword@cnu.ac.kr (S.W.J.); hoapt@cnu.ac.kr (T.H.P.); parkjinsong95@o.cnu.ac.kr (J.S.P.); chaeyeon05@o.cnu.ac.kr (C.Y.K.); chlkoala@naver.com (J.H.C.); yhk@cnu.ac.kr (Y.H.K.); 2 Subtropical/Tropical Organism Gene Bank, Jeju National University, Jeju 63243, Korea; 3 Drug & Disease Target Research Team, Division of Bioconvergence Analysis, Korea Basic Science Institute (KBSI), Cheongju 28119, Korea; heh4285@kbsi.re.kr

**Keywords:** impressic acid, anti-inflammation, Nrf2, AMPK, GSK3β

## Abstract

Inflammatory diseases are caused by excessive inflammation from pro-inflammatory mediators and cytokines produced by macrophages. The Nrf2 signaling pathway protects against inflammatory diseases by inhibiting excessive inflammation via the regulation of antioxidant enzymes, including HO-1 and NQO1. We investigated the anti-inflammatory effect of impressic acid (IPA) isolated from *Acanthopanax koreanum* on the lipopolysaccharide (LPS)-induced inflammation and the underlying molecular mechanisms in RAW264.7 cells. IPA attenuated the LPS-induced production of pro-inflammatory cytokines and reactive oxygen species, and the activation of the NF-κB signaling pathway. IPA also increased the protein levels of Nrf2, HO-1, and NQO1 by phosphorylating CaMKKβ, AMPK, and GSK3β. Furthermore, ML385, an Nrf2 inhibitor, reversed the inhibitory effect of IPA on LPS-induced production of pro-inflammatory cytokines in RAW264.7 cells. Therefore, IPA exerts an anti-inflammatory effect via the AMPK/GSK3β/Nrf2 signaling pathway in macrophages. Taken together, the findings suggest that IPA has preventive potential for inflammation-related diseases.

## 1. Introduction

Inflammation is a reaction to pathogens, irritants, or injury and is essential for protecting against bacterial and viral infections [1]. However, excessive inflammatory reactions increase the activation of macrophages and are a major cause of acute and chronic inflammatory diseases such as arthritis, atherosclerosis, and asthma [2,3]. Macrophages play an important role in immune defense and tissue regeneration by producing inflammatory mediators such as reactive oxygen species (ROS), nitric oxide (NO), prostaglandin E2 (PGE2), and pro-inflammatory cytokines [4]. By contrast, the unrestricted production and accumulation of pro-inflammatory mediators by macrophages promotes excessive inflammatory reactions, which adversely affect adjacent cells and tissues, leading to autoimmune diseases, chronic obstructive pulmonary disease (COPD), diabetes, and cancer [5,6].

Inflammatory responses induce macrophage differentiation into the M1 phenotype, thereby increasing the production of pro-inflammatory mediators and cytokines as well as improving immune defense and recovery from damage [4,7]. M2 macrophages produce anti-inflammatory mediators such as heme oxygenase-1 (HO-1) and NADPH dehydrogenase quinone 1 (NQO1). HO-1, which maintains cellular redox homeostasis, is expressed at a low levels in unstimulated normal cells [8,9]. However, HO-1 expression is increased by lipopolysaccharides (LPS), oxidative stress, pro-inflammatory cytokines, and transforming growth factor-β1 (TGF-β1) and protects cells from oxidative stress and inflammation [10,11]. HO-1 expression is regulated by the transcription factor nuclear factor-erythroid 2-related factor 2 (Nrf2), which modulates the expression of genes encoding phase II enzymes, including HO-1 and NQO1 [12]. Nrf2 in the cytosol is bound to the Kelch-like ECH associated protein1 (Keap1), from which it is separated by various factors and translocated to the nucleus, where it binds to the antioxidant response element (ARE), which increases the expression of phase II enzymes [13,14]. High levels of HO-1 and NQO1 suppress the inflammatory response in LPS-induced macrophages. In addition, the AMP-activated protein kinase (AMPK)/Nrf2/ARE signaling inhibits the LPS-induced increase in pro-inflammatory cytokines, such as TNF-α and IL-6, as well as the activation of the NF-κB signaling pathway [15].

AMPK, a key regulator of cellular metabolism, suppresses oxidative stress and inflammatory responses by activating antioxidant enzymes and its phosphorylation promotes the activity of Nrf2 by inducing glycogen synthase kinase 3 beta (GSK3β) phosphorylation at Ser-9 [16]. The phosphorylation of GSK3β at Ser-9 increases stability and induces the nuclear translocation and transcriptional activity of Nrf2 [17]. Liver kinase B1 (LKB1) and calcium/calmodulin-dependent protein kinase kinase beta (CaMKKβ), a factor upstream of AMPK, are associated with AMPK phosphorylation [18]. Numerous bioactive compounds derived from natural sources target the AMPK/Nrf2/ARE signaling pathway to exert antioxidant and anti-inflammatory effects. The increased activity of AMPK/Nrf2 signaling, induced by bioactive compounds, exerts an anti-inflammatory effect by increasing the expression of antioxidant enzymes, including HO-1 and NQO1 which suppresses NF-κB signaling [19,20,21]. Therefore, the elucidation of the mechanisms that regulate the interaction between AMPK and Nrf2 and the discovery of novel therapeutics will enhance the treatment of inflammatory diseases.

Impressic acid (IPA; 3α-11α-dihydroxylup-20(29)-en-28-oic acid) is a lupane-type triterpenoid isolated from *Acanthopanax koreanum*, which has been used as a traditional medicine for rheumatism, hepatitis, type 2 diabetes, and inflammatory disorders [22,23]. IPA downregulates matrix metalloproteinase-13 activity, which prevents cartilage destruction and increases eNOS activity and NO production, thereby inhibiting tumor necrosis factor alpha (TNF-α)-induced endothelial dysfunction [24,25]. IPA also enhances the activity of peroxisome proliferator-activated receptor γ (PPARγ) and attenuates TNF-α-induced NF-κB activity [26]. However, the anti-inflammatory effect of IPA on macrophages and its mechanism is unclear. Therefore, we investigated the effect of IPA on the inflammatory response and the underlying molecular mechanism in RAW264.7 cells.

## 2. Results

### 2.1. Cytotoxicity of IPA in RAW264.7 Cells

**Figure 1:**
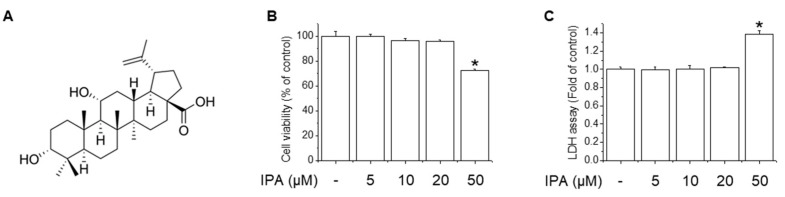
Effect of IPA on the cell viability and cytotoxicity of RAW264.7 cells. (**A**) Chemical structure of IPA. RAW264.7 cells were treated with 5–50 μM IPA for 24 h. Cell viability (**B**) and cytotoxicity (**C**) were measured by MTT and LDH assay. Data are the mean ± SD of three independent experiments. * Significantly different from the control at *p* < 0.01.

To investigate the cytotoxicity of IPA in RAW264.7 cells, we performed MTT and LDH assays. IPA at 50 μM showed significant cytotoxicity (Figure 1B,C). However, IPA did not show cytotoxicity at ≤20 μM. Therefore, cells were treated with up to 20 μM IPA in subsequent experiments.

### 2.2. Effect of IPA on Pro-Inflammatory Cytokine and ROS Levels

We investigated the effect of IPA on pro-inflammatory cytokine expression and NF-κB signaling in LPS-treated RAW264.7 cells. The experimental design of the co-treatment with IPA and LPS is described in Table 1.

**Figure 2:**
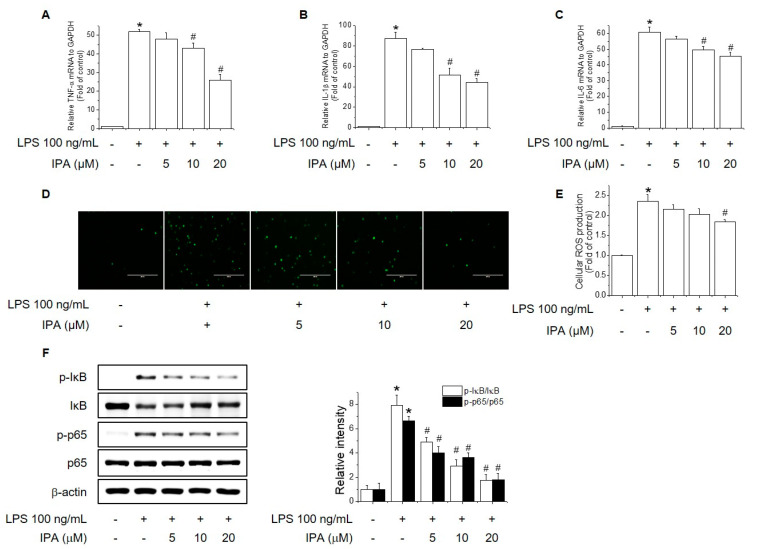
Effect of IPA on LPS-induced pro-inflammatory cytokine and ROS levels. (**A**–**C**) RAW264.7 cells were pretreated with 5–20 μM IPA for 12 h, 100 ng/mL LPS for 6 h, and TNF-α (**A**), IL-1β (**B**), and IL-6 (**C**) expression was analyzed by real-time PCR. (**D**,**E**) RAW264.7 cells were pretreated with 5–20 μM IPA for 12 h and 100 ng/mL LPS for 24 h. ROS levels measured by H_2_DCF-DA. (**F**) Effect of IPA on LPS-induced phosphorylation of IκB and NF-κB. RAW264.7 cells were pretreated with 5–20 μM IPA for 12 h followed by 100 ng/mL LPS for 1 h. * Significantly different from the control at *p* < 0.01. ^#^ Significantly different from the LPS treatment group at *p* < 0.01.

As shown in Figure 2A–C, pretreatment with IPA significantly reduced LPS-induced expression of the TNF-α, IL-1β, and IL-6 in RAW264.7 cells. IPA also attenuated the LPS-induced intracellular ROS level in a concentration-dependent manner (Figure 2D,E). Furthermore, IPA significantly inhibited LPS-induced phosphorylation of NF-κB and IκB in a concentration-dependent manner (Figure 2F). These results indicate that IPA exerts an anti-inflammatory effect by reducing the production of pro-inflammatory cytokines and ROS via the inhibition of NF-κB signaling in RAW264.7 cells.

### 2.3. Effect of IPA on Antioxidant Enzymes

**Figure 3:**
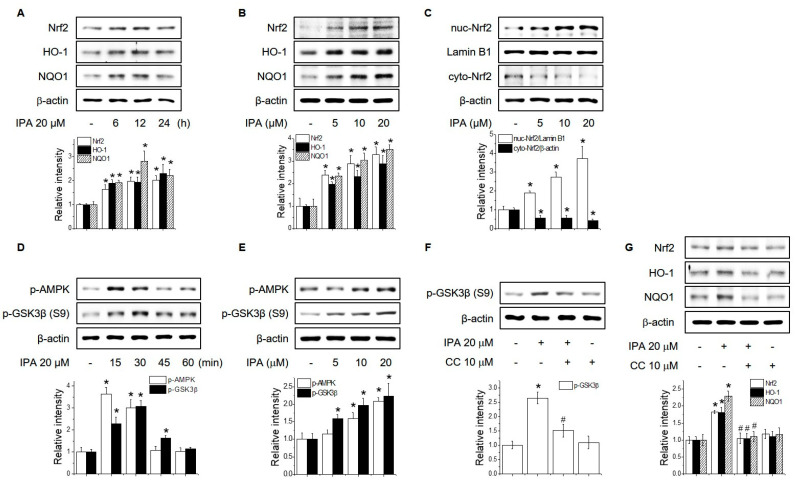
Effect of IPA on Nrf2 expression via the AMPK/GSK3β signaling pathway. RAW264.7 cells were treated with 20 μM IPA for 6–24 h (**A**) or 5–20 μM IPA for 12 h (**B**). The protein levels of Nrf2, HO-1, and NQO1 were determined by Western blotting. (**C**) Effect of IPA on Nrf2 translocation. Nuclear and cytosolic proteins of RAW264.7 cells were analyzed by Western blotting. RAW264.7 cells were treated with 20 μM IPA for 15–60 min (**D**) or 5–20 μM IPA for 30 min (**E**). The AMPK and GSK3β phosphorylation levels were determined by Western blotting. (**F**) RAW264.7 cells were pretreated with 10 μM compound C for 1 h, followed by 20 μM IPA for 30 min, and the GSK3β phosphorylation levels was determined by Western blotting. (**G**) RAW264.7 cells were cultured with 10 μM compound C for 1 h before the addition of 20 μM IPA for 12 h. * Significantly different from the control at *p* < 0.01. ^#^ Significantly different from the IPA treatment group at *p* < 0.01.

To evaluate the antioxidant effect of IPA on macrophages, we investigated the effect of IPA on the expression of Nrf2 and antioxidant enzymes in RAW264.7 cells. IPA significantly increased Nrf2, HO-1 and NQO1 expression in a time- and concentration-dependent manner (Figure 3A,B). In addition, IPA-treated RAW264.7 cells significantly enhanced the protein levels of Nrf2 in the nucleus, instead of decreasing it in the cytosol (Figure 3C). AMPK was reported to have exerted an anti-inflammatory effect by increasing the transcriptional activity of Nrf2 via the inhibition of GSK3β. As shown in Figure 3D,E IPA induced AMPK phosphorylation and inactivated GSK3β via Ser9 phosphorylation. Pretreatment with compound C, an AMPK inhibitor, suppressed IPA-induced GSK3β via Ser9 phosphorylation and protein levels of Nrf2, HO-1, and NQO1 (Figure 3F,G). These data suggested that IPA enhances GSK3β-mediated Nrf2 activity by inducing phosphorylation of AMPK and increasing HO-1 and NQO1 expression in RAW264.7 cells.

### 2.4. Role of CaMKKβ in IPA-Mediated Increased Nrf2 Expression via AMPK/GSK3β Signaling

**Figure 4:**
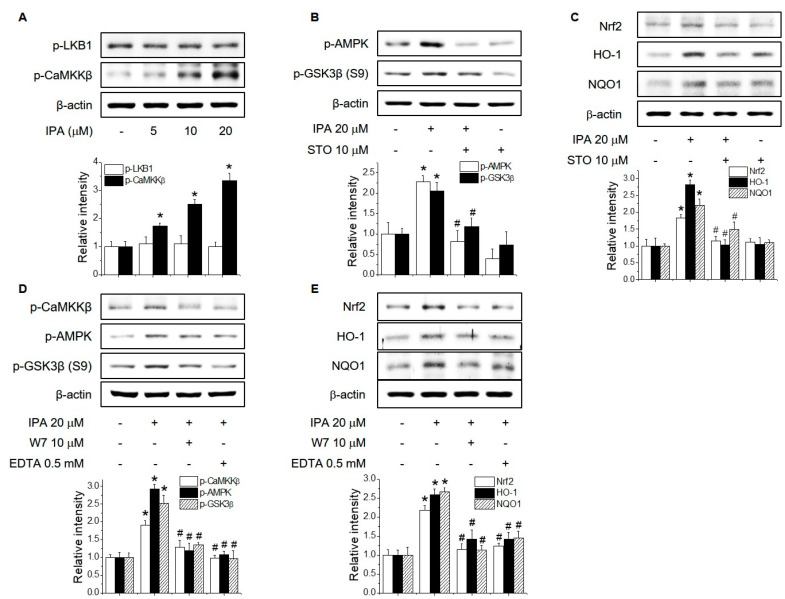
Role of CaMKKβ in IPA-induced Nrf2 expression via the AMPK/GSK3β signaling pathway. (**A**) RAW264.7 cells were treated with 5–20 μM IPA for 15 min. LKB1 and CaMKKβ phosphorylation levels were determined by Western blotting. (**B**) RAW264.7 cells were cultured with 10 μM STO-609 for 1 h before adding 20 μM IPA for 30 min. AMPK and GSK3β phosphorylation levels were determined by Western blotting. (**C**) RAW264.7 cells were cultured with 10 μM STO-609 for 1 h before adding 20 μM IPA for 12 h. The protein levels of Nrf2, HO-1, and NQO1 were determined by Western blotting. (**D**) RAW264.7 cells were pretreated with 10 μM W7 or 0.5 mM EDTA for 1 h, followed by 20 μM IPA for 15 min. CaMKKβ, AMPK, and GSK3β phosphorylation levels were determined by Western blotting. (**E**) RAW264.7 cells were cultured with 10 μM W7 or 0.5 mM EDTA for 1 h before adding 20 μM IPA for 12 h. * Significantly different from the control at *p* < 0.01. ^#^ Significantly different from the IPA treatment group at *p* < 0.01.

We hypothesized that the anti-inflammatory effect of IPA may be due to the activation of AMPK via LKB1 or CaMKKβ. The activation of AMPK by LKB1 and CaMKKβ is involved in the regulation of the inflammatory response [18]. To investigate whether LKB1 and CaMKKβ are required for IPA-induced Nrf2 expression via the AMPK/GSK3β signaling pathway, we examined the effect of IPA on the phosphorylation of LKB1 and CaMKKβ. As shown in Figure 4A, IPA significantly increased the phosphorylation of CaMKKβ but did not cause phosphorylation of LKB1 in the RAW264.7 cells. Moreover, pretreatment with STO-609, a CaMKKβ inhibitor, suppressed the IPA-mediated increased phosphorylation of GSK3β and AMPK, and inhibited the IPA-induced protein levels of Nrf2, HO-1, and NQO1 (Figure 4B,C). The activation of CaMKKβ by calcium influx regulates the inflammatory response via AMPK phosphorylation. To determine whether calcium signaling is required for IPA-induced Nrf2 expression via AMPK/GSK3β signaling, we blocked calcium signaling using the calmodulin antagonist W7 and EDTA prior to treatment with IPA in the RAW264.7 cells. W7 and EDTA suppressed the IPA-induced phosphorylation of GSK3β, AMPK, and CaMKKβ and the expression of Nrf2, HO-1, and NQO1 (Figure 4D,E). These results suggested that CaMKKβ promotes Nrf2 expression and antioxidant enzyme expression via AMPK/GSK3β signaling in response to IPA.

### 2.5. Role of Nrf2 in the Inhibitory Effect of IPA on Pro-Inflammatory Cytokines and the NF-κB Signaling Pathway

**Figure 5:**
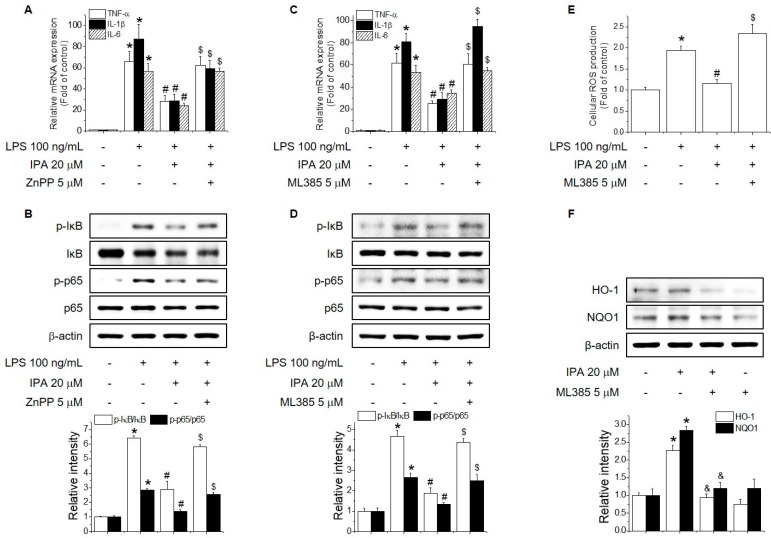
Role of Nrf2 in the inhibitory effect of IPA on LPS-induced inflammation. (**A**,**C**) RAW264.7 cells were cultured with 5 μM ZnPP or ML385 for 1 h before adding 20 μM IPA for 12 h, followed by 100 ng/mL LPS for 6 h. TNF-α, IL-1β, and IL-6 expression were analyzed by real-time PCR. (**B**,**D**) RAW264.7 cells were cultured with 5 μM ZnPP or ML385 for 1 h before adding 20 μM IPA for 1 h, followed by 100 ng/mL LPS for 1 h. The phosphorylation levels of IκB and NF-κB were determined by Western blotting. (**E**) RAW264.7 cells were cultured with 5 μM ZnPP or ML385 for 1 h before adding 20 μM IPA for 12 h, followed by 100 ng/mL LPS for 24 h. The ROS levels was assayed using H_2_DCF-DA. (**F**) RAW264.7 cells were cultured with 5 μM ML385 for 1 h before adding 20 μM IPA for 12 h. * Significantly different from the control at *p* < 0.01. ^#^ Significantly different from the LPS treatment group at *p* < 0.01. ^$^ Significantly different from the LPS and IPA treatment group at *p* < 0.01. ^&^ Significantly different from the IPA treatment group at *p* < 0.01.

Nrf2/HO-1 signaling plays a key role in regulating inflammation by decreasing oxidative stress and catalyzing the heme reaction to regulate NF-κB signaling via the production of carbon monoxide [27]. IPA lowered the increased mRNA levels of TNF-α, IL-1β, and IL-6, whereas pretreatment with ZnPP, an HO-1 inhibitor, significantly reversed the inhibitory effect of IPA on the mRNA levels of TNF-α, IL-1β, and IL-6 (Figure 5A). In addition, ZnPP attenuated the inhibitory effect of IPA on LPS-induced phosphorylation of NF-κB and IκB (Figure 5B). Furthermore, ML385 reversed the inhibitory effect of IPA on the increased mRNA levels of TNF-α, IL-1β, and IL-6, as well as the phosphorylation of NF-κB and IκB (Figure 5C,D). Because it is an Nrf2 inhibitor, ML385 also reversed the inhibitory effect of IPA on LPS-induced ROS production and HO-1 and NQO1 expression (Figure 5E,F). These data suggest that IPA exerts an anti-inflammatory effect by promoting the production of antioxidant enzymes via the Nrf2 signaling pathway.

## 3. Discussion

Inflammation is an innate immune response to protect tissues and cells from damage and infection. However, an excessive inflammatory response causes inflammatory diseases by increasing the production and release of pro-inflammatory agents such as ROS and pro-inflammatory cytokines [28,29]. Lupane-type triterpenoids, including IPA, from the leaves of *A. koreanum* have shown protective effects against excessive inflammation, endothelial dysfunction, and cartilage destruction [23,24,25]. However, the anti-inflammatory effect and molecular mechanism are unclear. In this study, IPA suppressed LPS-induced inflammatory responses by regulating the AMPK/GSK3β/Nrf2 signaling pathway in RAW264.7 macrophages.

Macrophages conduct phagocytosis, antigen presentation, and secretion of pro-inflammatory cytokines in response to LPS [30]. An excessive inflammatory response caused by macrophage activation can lead to autoimmune diseases, type 1 diabetes, and chronic obstructive pulmonary disease as a result of the uncontrolled production of pro-inflammatory cytokines [31]. In this study, we demonstrated that IPA attenuated LPS-induced production of the pro-inflammatory cytokines TNF-α, IL-1β, and IL-6 as well as the generation of ROS in RAW264.7 cells. IPA also reduced LPS-induced phosphorylation of NF-κB and IκB. Bioactive compounds ameliorate LPS-induced inflammation by inhibiting NF-κB signaling via a decrease in the expression of inflammatory mediators [32]. In addition, the regulation of anti-oxidant enzymes by Nrf2 activity is closely related to the anti-inflammatory effects of reducing oxidative stress such as ROS [33]. Therefore, the anti-inflammatory effect of IPA involves suppression of NF-κB signaling.

Nrf2 is a key signaling pathway that protects against inflammation by regulating the expression of antioxidant enzymes, including HO-1 and NOQ1 [34]. In particular, HO-1 exerts an anti-inflammatory effect by inhibiting NF-κB signaling via the production of carbon monoxide, a metabolite of the heme reaction [27]. In this study, we found that IPA increased the protein levels of Nrf2, HO-1, and NQO1 and induced the nuclear translocation of Nrf2 in RAW264.7 cells. Furthermore, the inhibition of Nrf2 and HO-1 suppressed the inhibitory effect of IPA on LPS-induced pro-inflammatory cytokine expression, ROS production, and the NF-κB signaling pathway. These findings suggest that IPA protects against inflammation by upregulating antioxidant enzymes via the Nrf2 signaling pathway. The AMPK signaling pathway mediates Nrf2 activation, thereby activating the expression of antioxidant enzymes [35]. Furthermore, the phosphorylation of AMPK inhibits GSK3β by phosphorylation at Ser9 and promotes the stability of Nrf2 by blocking its proteolytic degradation [17,36]. Our results showed that IPA increased the phosphorylation of AMPK and GSK3β. Furthermore, AMPK inhibition suppressed the phosphorylation of GSK3β and the protein levels of Nrf2, HO-1, and NQO1 that were induced by IPA. Ci et al. reported that betulin, which has a similar chemical structure to IPA, exerts an anti-inflammatory effect via the AMPK/GSK3β/Nrf2 signaling pathway. This is consistent with our findings, [37] which suggest that IPA may exert the protective effects on excessive inflammation response by regulating anti-oxidant enzymes via the AMPK/GSK3β/Nrf2 signaling pathway.

The upstream kinases for AMPK activity are important for regulating the inflammatory response. LKB1 and CaMKKβ regulate the activity of AMPK by phosphorylation [38]. LKB1 modulates the phosphorylation of AMPK in response to changes in the ATP-to-ADP ratio, and CaMKKβ increases the phosphorylation of AMPK in response to increasing intracellular calcium [39]. In this study, IPA phosphorylated CaMKKβ, but not LKB1, in RAW264.7 cells. In addition, the inhibition of CaMKKβ attenuated the IPA-induced phosphorylation of AMPK and GSK3β, as well as the protein levels of Nrf2, HO-1, and NQO1. Furthermore, the blocking of calcium signaling suppressed the IPA-induced expression of antioxidant enzymes and the phosphorylation of CaMKKβ, AMPK, and GSK3β. CaMKKβ activation by calcium signaling exerts anti-inflammatory and neuroprotective effects via the AMPK/GSK3β/Nrf2 pathway [40,41]. Therefore, CaMKKβ mediates AMPK-induced Nrf2 activation by IPA in macrophages. Taken together, these findings indicate that IPA may act as a potential natural anti-inflammatory treatment because of its effect on antioxidant enzymes via the AMPK/GSK3β/Nrf2 signaling pathway.

**Figure 6:**
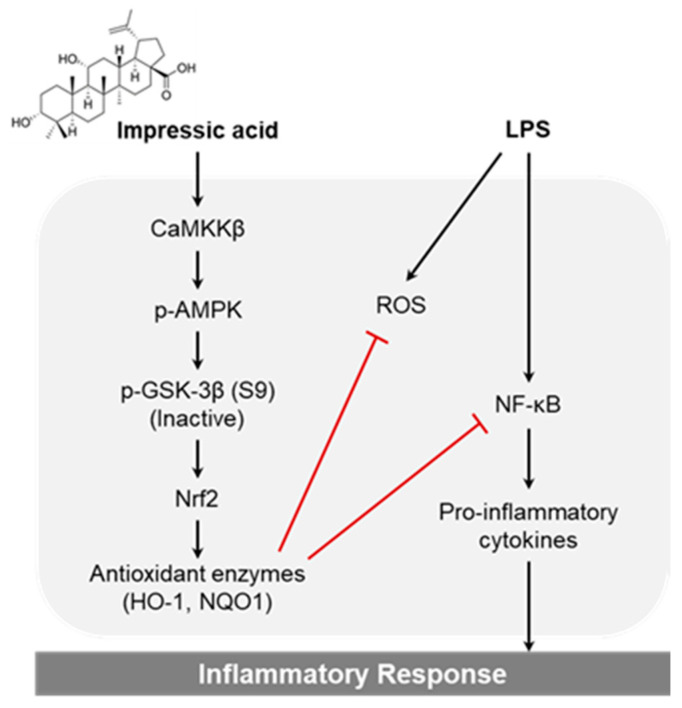
Proposed mechanism of the anti-inflammatory effect of IPA via the AMPK/GSK3β/Nrf2 signaling pathway in RAW264.7 macrophages. IPA increased the activity of CaMKKβ by increasing the intracellular calcium levels and activating the phosphorylation of AMPK and GSK3β, leading to increased protein levels of Nrf2, HO-1, and NQO1. Consequently, IPA attenuated LPS-induced inflammation by promoting the production of antioxidant enzymes via the AMPK/GSK3β/Nrf2 signaling axis.

In conclusion, we established that IPA exhibits anti-inflammatory effects by attenuating pro-inflammatory cytokines expression in LPS-induced macrophages. The anti-inflammatory effects of IPA were mediated by the novel activity of antioxidant enzymes via the AMPK/GSK3β/Nrf2 axis (Figure 6). Therefore, we suggest that IPA is a valuable as a potential candidate for use in the prevention of various inflammatory diseases.

## 4. Materials and Methods

### 4.1. Chemicals and Reagents

IPA (Figure 1A) was provided by Dr. Young-Ho Kim (Chungnam National University, Daejeon, Korea). Dulbecco’s modified Eagle’s medium (DMEM), fetal bovine serum (FBS), penicillin–streptomycin, and trypsin were purchased from Welgene (Gyeongsan, Korea). STO-609, zinc protoporphyrin IX (ZnPP), ML385, and LPS were purchased from Sigma-Aldrich (St. Louis, MO, USA). Compound C were obtained from Tocris (Cookson, Bristol, UK), and EDTA was purchased from GenDEPOT (Barker, TX, USA). W7 was obtained from Calbiochem (La Jolla, CA, USA). Antibodies against p-NF-κB p65, p-IκB, IκB, p-AMPK, p-CaMKKβ, p-GSK3β(Ser9), and p-LKB1 were from Cell Signaling Technology (Beverly, MA, USA). Antibodies against NF-κB p65, HO-1, NOQ1, Nrf2, and β-actin were purchased from Santa Cruz Biotechnology (Santa Cruz, CA, USA). Lamin B1 was purchased from Bioss Antibody, Inc. (Woburn, MA, USA). Tetrazole 3-(4,5-dimethylthiazol-2-yl)-2,5-diphenyltetrazolium bromide (MTT) was acquired from USB Corporation (Cleveland, OH, USA). The cytotoxicity assay kit was obtained from Roche Applied Science (Indianapolis, IN, USA). All other chemicals were of the highest grade commercially available.

### 4.2. Cell Culture and Cell Viability Assay

RAW 264.7 mouse macrophages were obtained from the American Type Culture Collection (Bethesda, MD, USA) and cultured in DMEM (containing 10% FBS, 100 U/mL penicillin, and 100 μg/mL streptomycin) in a 37 °C incubator with 5% CO_2_. Cells in 48-well plates were treated with IPA (5, 10, 20, and 50 μM) for 24 h. Next, an MTT solution was added, followed by incubation for 30 min, and formazan crystals were solubilized by adding DMSO. The absorbance at 550 nm was measured using a BioTek Synergy HT microplate reader (BioTek Instruments, Winooski, VT, USA). Medium was collected for lactate dehydrogenase assay, and the absorbance at 490 nm was measured using a BioTek Synergy HT microplate reader (BioTek Instruments). Cell viability (%) and cytotoxicity (fold-change) were quantified based on the absorbance of treated cells relative to the control cells (exposed to DMSO alone).

### 4.3. RNA Extraction and Quantitative Reverse Transcriptase-Polymerase Chain Reaction

Total RNA was extracted from RAW264.7 cells using RNAiso Plus Total RNA Extraction Reagent (TaKaRa, Shiga, Japan), and cDNA was synthesized using the BioFact RT Series Kit. Quantitative reverse transcriptase-polymerase chain reaction (qRT-PCR) was performed to analyze the expression of pro-inflammatory cytokines with continuous monitoring using Bio-Rad CFX Connect Real-Time PCR software, version 1.4.1 (Bio-Rad Laboratories, Hercules, CA, USA). The following primers were used: IL-1β forward, 5′-GAAGCGCTGCTTCCAAACCT-3′, IL-1β reverse, 5′-TGATGTGCTGCTGCGAGATT-3′, TNF-α forward, 5′-ACCGTCAGCCGATTTGCTAT-3′, TNF-α reverse, 5′-CTGGAAGACTCCTCCCAGGT-3′, IL-6 forward, 5′-TACCACTTC- ACAAGTCGGAGGC-3′, IL-6 reverse, 5′-CTGCAAGTGCATCATCATCGTTGTTC-3′. Expression levels were normalized to those of GAPDH.

### 4.4. Western Blotting

RAW264.7 cells were harvested and lysed with CETi Lysis Buffer (TransLab, Daejeon, Korea) for 30 min to extract total protein and centrifuged at 13,000 rpm for 15 min. Nuclear and cytoplasmic proteins were isolated using nuclear extract kits (Active Motif, Carlsbad, CA, USA). The supernatant was collected, and the protein concentration was determined at 595 nm using a Pro-Measure protein assay kit (Intron Biotechnology, Seongnam, Korea). Equal amounts of total cellular proteins were separated by sodium dodecyl sulfate polyacrylamide gel electrophoresis (SDS-PAGE) and transferred onto nitrocellulose membranes. After blocking with 5% skim milk, blots were incubated with the primary antibodies overnight at 4 °C followed by the corresponding secondary antibodies. Protein bands were visualized using a Hisol ECL Plus Detection Kit (BioFact, Daejeon, Korea). ImageJ software (NIH, Bethesda, MD, USA) was used to calculate the integrated optical densities of protein bands, which were normalized to that of the internal control.

### 4.5. ROS Assay

ROS production in RAW 264.7 cells was measured using the redox-sensitive fluorescent dye H_2_DCF-DA. After being treated with IPA and LPS, the upper medium in each well of 48-well plates was discarded, and the cells were probed with 2 μM H_2_DCFDA at 37 °C for 30 min and rinsed twice with PBS. The fluorescence intensity as an indication of the ROS levels was measured using a BioTek Synergy HT microplate reader (BioTek Instruments; excitation, 490 nm; emission, 530 nm).

### 4.6. Statistical Analysis

Data are the mean ± standard deviation of at least three independent experiments. Statistical analysis was performed by one-way analysis of variance. The Newman–Keuls test was used for comparisons of multiple groups. Statistical significance was defined as *p* < 0.01.

## Figures and Tables

**Table 1:** Experimental settings of co-treatment IPA and LPS.

	Pre-Treatment (IPA)	Post-Treatment (LPS)	Target
Gene expression	12 h	6 h	TNF-α, IL-1β, IL-6
Phosphorylated protein expression	12 h	1 h	p-IκB, p-NF-κB
ROS generation	12 h	24 h	ROS

## Data Availability

The data presented in this study are available on request from the corresponding author.

## Abbreviations

ROS	reactive oxygen species
HO-1	heme oxygenase-1
NQO1	NADPH dehydrogenase quinone 1
LPS	lipopolysaccharide
Nrf2	nuclear factor-erythroid 2-related factor 2
ARE	antioxidant response element
Keap1	Kelch-like ECH associated protein1
AMPK	AMP-activated protein kinase
GSK3β	glycogen synthase kinase 3 beta
LKB1	liver kinase B1
CaMKKβ	calcium/calmodulin-dependent protein kinase kinase beta
IPA	impressic acid
NF-κB	nuclear factor kappa B
IκB	inhibitor of nuclear factor kappa B
TNF-α	tumor necrosis factor alpha
IL-1β	interleukin 1 beta
IL-6	interleukin-6
CC	compound C
EDTA	ethylenediaminetetraacetic acid
ZnPP	zinc protoporphyrin IX
